# An efficient system matrix factorization method for scanning diffraction based strain tensor tomography

**DOI:** 10.1107/S2053273323008136

**Published:** 2023-09-29

**Authors:** Axel Henningsson, Stephen A. Hall

**Affiliations:** a Division of Solid Mechanics, Lund University, Ole Römersväg 1, Lund, Sweden; STFC Rutherford Appleton Laboratory, United Kingdom

**Keywords:** X-ray diffraction, strain tensor, tomography, diffraction imaging

## Abstract

Diffraction-based tomographic strain tensor reconstruction problems in which a strain tensor field is determined from measurements made in different crystallographic directions are considered in the context of sparse matrix algebra. Previous work has shown that the estimation of the crystal elastic strain field can be cast as a linear regression problem featuring a computationally involved assembly of a system matrix forward operator. This operator models the perturbation in diffraction signal as a function of spatial strain tensor state. The structure of this system matrix is analysed and a block-partitioned factorization is derived that reveals the forward operator as a sum of weighted scalar projection operators. Moreover, the factorization method is generalized for another diffraction model in which strain and orientation are coupled and can be reconstructed jointly. The proposed block-partitioned factorization method provides a bridge to classical absorption tomography and allows exploitation of standard tomographic ray-tracing libraries for implementation of the forward operator and its adjoint. Consequently, RAM-efficient, GPU-accelerated, on-the-fly strain/orientation tensor reconstruction is made possible, paving the way for higher spatial resolution studies of intragranular deformation.

## Abstract 2

Matrix analysis is used to provide a computationally efficient mathematical framework for diffraction-based strain tomography.

## 1. Introduction

Diffraction-based strain tomography is an experimental technique deployed for estimation of the six-component elastic strain tensor field, 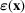 , within the bulk of polycrystalline aggregates. Whether used with X-rays (Hektor *et al.*, 2019 ▸; Korsunsky *et al.*, 2005 ▸; Lionheart & Withers, 2015 ▸) or neutrons (Hendriks *et al.*, 2020 ▸), the method offers a unique possibility to probe the internal heterogeneity of the strain in dense materials in a non-destructive way. In essence, the measured diffraction signal from the specimen can be reduced to average strains along line integral domains across a sample volume. Each of these scalar strain measures, 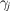 , can be associated to a spatial sampling direction, 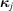 , which in general varies between measurements. Considering a set of 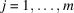 such measurements, it is possible to construct a global linear system of equations, 

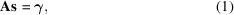

where 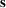 holds the basis coefficients of a decomposed strain tensor field and 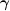 is a vector with all measurements [the formation of 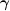 from raw diffraction images and the decoupling of the crystal strain from orientation are discussed by Henningsson & Hendriks (2021 ▸)]. The rows of the system matrix, 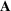 , are required to contain the integral weights of the strain tensor basis functions combined with non-linear combinations of the components of 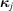 . Using measurements only from a single axis of rotation, there exist no known, closed-form, direct back-projection algorithms to recover the strain tensor field 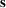 . This motivates the need for iterative solvers, which may seem to require the assembly of 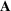 . Unfortunately, the storage of 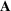 can be very RAM inefficient and the assembly routines needed to construct 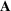 involve ray-tracing through the strain tensor volume. Indeed, direct storage of the forward operator is unfeasible for high-resolution scalar, absorption-based, tomography. Considering a fixed resolution, strain tomography of symmetric strain tensor fields offers no relaxation in this respect as the number of non-zeros in the system matrix, 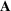 , is a factor six greater compared with scalar tomography. As a result, several existing reconstruction methods have been cast in settings with few strain tensor basis functions limiting the achievable reconstruction resolution (Henningsson *et al.*, 2020 ▸; Henningsson & Hendriks, 2021 ▸; Hendriks *et al.*, 2020 ▸). Similarly, in scanning 3D X-ray diffraction (scanning-3DXRD) microscopy applications, it is common to collapse the rich 2D pixel intensity distribution of the recorded diffraction peaks to a single centre of gravity prior to the pursuit of strain reconstruction (Hayashi *et al.*, 2015 ▸).

We present a system matrix factorization for strain tensor tomography in which the forward operator, 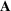 , can be implemented as a weighted sum of scalar forward projections as 

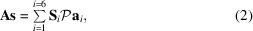

where 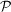 is a scalar forward projection operator, 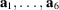 are the six individual components of the strain tensor field and 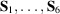 are diagonal weight matrices. This factorization allows for RAM-efficient, on-the-fly implementations to be easily achieved with existing tomographic libraries (for scalar projection). Additionally, our proposed factorization allows for access to GPU-accelerated implementations commonly deployed in scalar tomography to facilitate large sparse iterative solvers (Palenstijn *et al.*, 2011 ▸; van Aarle *et al.*, 2015 ▸, 2016 ▸).

For illustrative purposes we have selected to present our derivations in the context of strain reconstruction and for the experimental setup of scanning-3DXRD. The methodology is, however, also applicable for other neutron and X-ray scanning diffraction experiments given that a fixed axis of rotation is used and that the diffraction peak centre-of-mass positions can be accurately measured (typically in far-field geometry). The key ingredient in our derivation is the linearity of the diffraction model, which allows us to rearrange the order of the involved operators. In contrast to the far-field diffraction setting considered in this paper, near-field diffraction methods (Reischig & Ludwig, 2020 ▸) model the full detector intensity distribution of the diffraction peaks rather than the peak centroid positions. As a result, the forward operator in near-field diffraction models depends non-linearly on the intragranular strain and orientation. To highlight that our factorization method is applicable to multiple models, as long as they fall within the class of far-field diffraction, we derive and demonstrate (in Appendix *C* [App appc]) a factorization similar to that of equation (2) for a previously suggested diffraction model that features coupling between the intragranular strain and orientation. This factorization enables efficient reconstruction of the full intragranular deformation field, including both strain and orientations.

## 2. Per-ray factorization

Given an unknown, symmetric, second-order strain tensor field, 

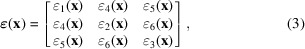

defined on a 3D spatial domain, 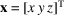 , we shall consider measurements of the average strain, 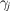 , on the line integral domain 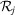 as 

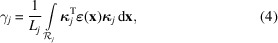

where 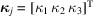 is a unit normal vector that describes the sampled strain direction and 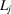 is the ray intersection path length measured over the compact support of 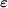 . For scanning-3DXRD the formation of 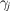 from the raw diffraction image data has been described elsewhere (Henningsson & Hendriks, 2021 ▸). Following a flattened vector format similar to that of Henningsson & Hendriks (2021 ▸) we find the alternative measurement model 

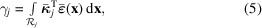

where 

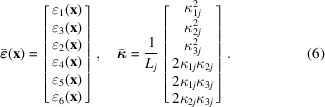

Let 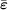 be decomposed on 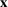 with *n* basis functions 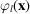 as 

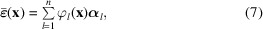

where the basis coefficients 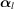 are defined as 

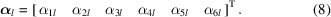

In the following we select 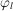 to represent an equidistant grid of pixels such that 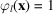 when 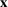 is in pixel number *l* and 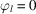 otherwise. By insertion of (7) into (5) we have 

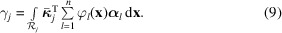

Reordering the integral and sum we can write 

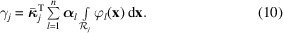

We now introduce the vector 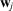 which contains the scalar weights of the ray integral with respect to basis functions, 

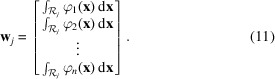

Using the weights, 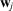 , we may form a matrix projection operator that projects the six components of the strain field along a single ray path as 

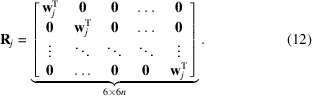

Additionally we introduce the vectors 

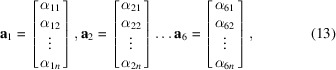

and stack the basis coefficients of the unknown strain tensor field in a single column vector as 

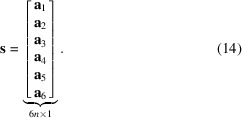

We can now facilitate a fully vectorized and discretized format of the measurement model, equation (4), as 

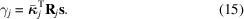

To arrive at a global format, in which several measurements, 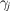 , are considered simultaneously, we introduce the vector 

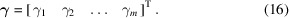

Stacking the matrices 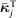 and 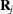 in the same fashion, 

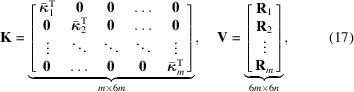

we find the global matrix formulation as 

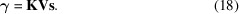

We note that in equation (18) the matrix 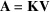 is factorized in two terms: 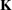 , which contains information on the directional sampling of the strain field, and 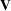 , which holds information on the projections of the sampled fields.

## 3. Hexa-block-diagonal form

In scalar tomography the forward projection operator, 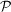 , is commonly block-partitioned over a series of projection views, 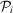 , as 

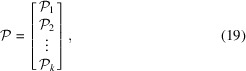

where each projection view, 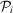 , represents an ordered set of parallel line integrals defined over a single scalar field. In contrast, we note that the ray integrals contained in 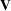 , as defined by equations (12) and (17), are neither ordered in complete views nor defined over a single scalar field. We therefore seek to reorder and partition the rays in 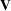 in a way that will allow our projection operator to be easily implemented using standard tomographic libraries. To this end, we note that the set of rows in 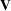 separated by a fixed multiple 6, with start at row number 1, forms the block-partitioned matrix 

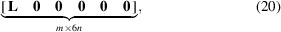

where 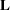 is now acting on the single scalar field 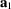 . If the measurements in 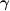 are selected to be stacked in complete projection views, we find that 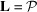 . Since the initial selected ordering of measurements in 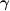 is arbitrary, we shall assume that this ordering has been selected. Now, by simply repeating the row shifting operation with increasing row starting index, 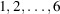 , it is possible to mutate 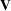 into the block-diagonal matrix form, 

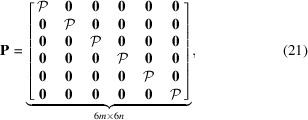

which contains the reordered rows of 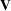 . Naturally, to maintain the global formulation in equation (17), we are required to now also modify 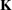 . The shifting of the rows of 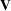 , therefore, requires a corresponding shifting of columns in 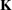 , leading to the block-partitioned matrix 

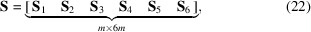

with diagonal blocks 

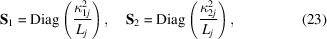




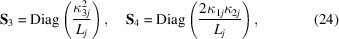




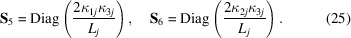

It is now possible to write 

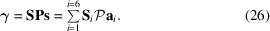

In this factorization the execution of the forward operator, 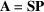 , corresponds to six scalar forward projections followed by the application of 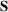 , which, due to its diagonal form, presents a modest 6*m* multiplications and additions. The implementation of 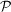 can be directly achieved by any ray-tracing library, *e.g.* the *ASTRA-toolbox* (Palenstijn *et al.*, 2011 ▸; van Aarle *et al.*, 2015 ▸, 2016 ▸). The implementation of 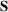 is trivial and, owing to its diagonal form, there is no need to assemble the matrix, as it suffices to store the six vectors of diagonal weights. Since the six projections being executed in 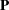 are independent, we note that the resulting arrays, 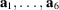 , may be stacked and projected in parallel on a GPU. To service the diffraction imaging community, and to illustrate how equation (26) can be put to use to achieve an easily implementable GPU-accelerated diffraction model, we provide an open-source demo Python code at https://github.com/AxelHenningsson/flyxdm.

## 4. Generalizations

For the sake of clarity, we derived equation (26) in the setting of strain reconstruction. This setting features scalar measurements, 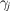 , which simplifies the exposition and allows us to focus on the core rearrangement of equations necessary to arrive at our block-partitioned factorized format. The same algebraic manipulations can be used to factorize a wider class of linear far-field diffraction models. We demonstrate the generality of our matrix factorization method in Appendix *C* [App appc] where we have pursued an extended diffraction model originally suggested by Henningsson *et al.* (2020 ▸). In this alternative setting the intragranular orientation field is jointly reconstructed with the strain tensor field and the measurement associated to the ray integral is vector valued rather than scalar.

To reconstruct a target field, 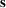 , in practice, it is often desirable to introduce a measurement weight matrix, 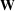 , that describes the measurement precision. For instance, in the work of Henningsson *et al.* (2020 ▸) ▸ a diagonal weight matrix was used to reconstruct strain in a weighted least-squares sense. We note that our factorization is indifferent to the introduction of 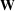 and the global equation system would in practical application be extended as 

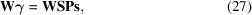

where 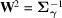 is the covariance of the measurements, 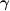 .

Another practical concern is the incorporation of constraints on the solution vector, 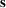 . One popular approach is to modify the basis of the unknown target field to encode the prior knowledge. To exploit our factorized format in these settings, we suggest introducing a rendering matrix, 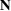 , that maps the basis coefficients, 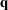 , from the constrained basis set back to the pixel basis coefficients, 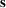 , as 

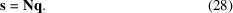

The resulting global equations now become 

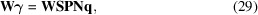

where the columns of 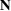 can be interpreted as pixel images of the selected basis set. As the forward operator, 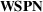 , and the adjoint operator, 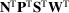 , still feature the desired multiplicative block-partitioned split between 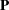 and 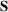 , we conclude that the results in equation (26) can be exploited in a wide range of applications.

As a final note, on the topic of generalizations, we would like to mention that, just as the tensor components of the target field can be stacked into a 3D volume and projected in parallel on a GPU card, one may instead consider stacking grain slices into a volume and projecting each tensor component separately. This modification, reconstructing a full grain volume rather than a grain slice, has no impact on the algebraic format of equation (26). The rendering matrix, 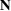 , is then computing the coefficients of a set of voxels that are projected as a 3D volume by 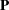 .

## 5. Demonstration

To demonstrate the memory benefits that can be achieved using equation (26) compared with assembling and storing the sparse matrix, 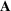 , we consider a single-crystal diffraction simulation case study. The simulation is described in detail in Appendix *A* [App appa], together with illustrations of the reconstructions achieved when exploiting the format of equation (26) during regression (Appendices *B* [App appb] and *C* [App appc]). The supplementary code used to generate the simulation data as well as the reconstructions is openly available at https://github.com/AxelHenningsson/flyxdm.

**Figure 1:**
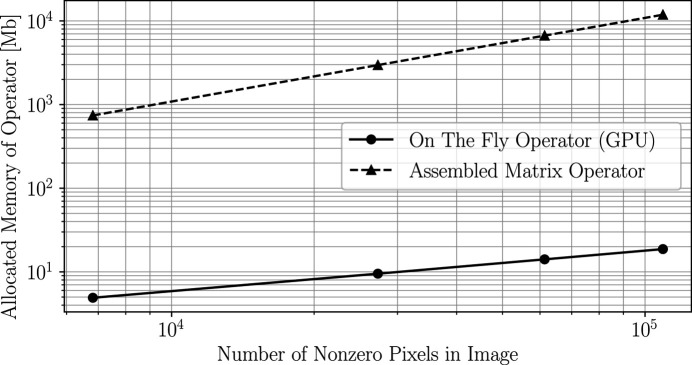
Number of megabytes of computer RAM necessary to compute 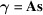 using either an assembled sparse matrix (dashed line) or, alternatively, the discussed factorization in conjunction with an on-the-fly projection operator, 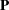 (solid line). Note that the benchmarks correspond to a single-crystal grain slice and 500 diffraction peaks (projection views) as described in Appendix *A* [App appa].

In Fig. 1 ▸ we present the number of megabytes of computer RAM necessary to compute 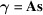 using either a fully assembled sparse matrix 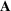 or, alternatively, the factorization 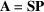 , where 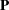 is represented using pre-existing, on-the-fly, projection operators, available in the *ASTRA-toolbox* (van Aarle *et al.*, 2015 ▸). Considering that the results presented in Fig. 1 ▸ represent the reconstruction of a single grain slice using 500 projection views (each corresponding to a diffraction event), it is evident that parallel, high-resolution, full volume/sample reconstructions are unfeasible using an assembled format of 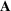 . For instance, reconstructing, in parallel, a single, cubic-shaped grain volume, with a cross-sectional resolution of 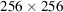 pixels from ∼300 unique (with respect to Miller index) diffraction peaks would require ∼1 TB of computer RAM storage.

## 6. Conclusion

We have presented a system matrix factorization for strain tensor tomography in which the directional sampling of the strain tensor field is separated from the tomographic projection operator. The proposed format allows for the exploitation of standard tomographic ray-tracing libraries in the implementation of the forward operator. We have also shown how our factorization method can be generalized for other diffraction models, for example one in which strain and orientation are coupled. We have provided an openly available GPU implementation of the approach and demonstrated the computational efficiency of our factorization method through application to a model example. By enabling RAM-efficient, GPU-accelerated, on-the-fly strain/orientation tensor reconstruction, our results facilitate higher spatial resolution studies of intragranular deformation.

## Figures and Tables

**Figure 2:**
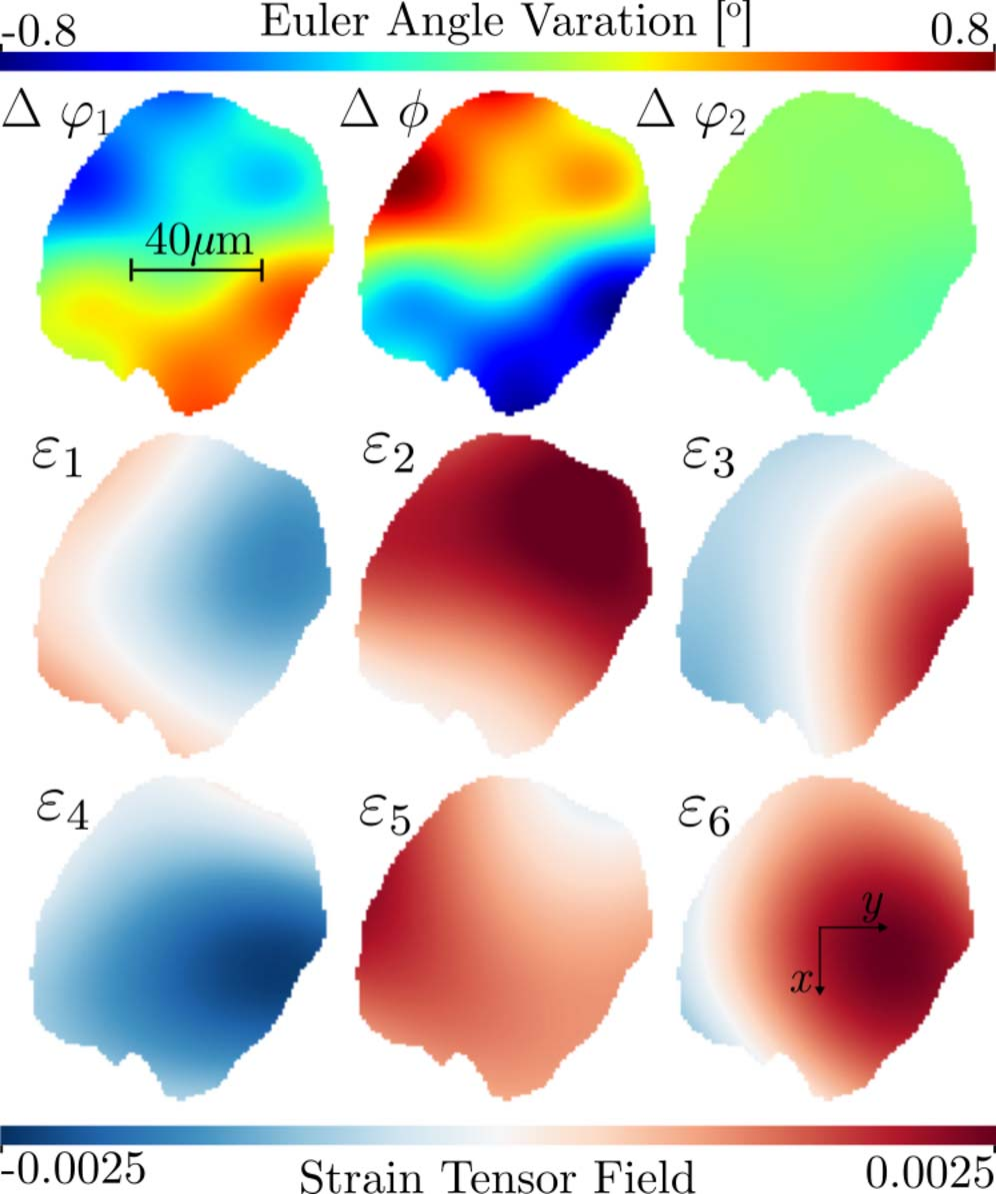
Strain (bottom) and mosaicity (top) in a simulated 2D grain slice of α-quartz (SiO_2_). The Bunge Euler angles are displayed as variations around their respective mean values.

**Figure 3:**
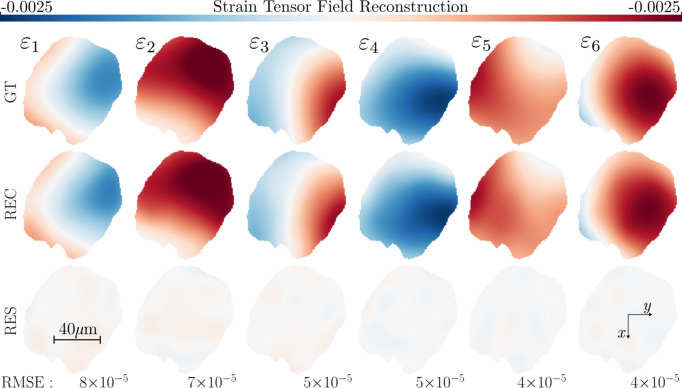
Strain tensor reconstruction (REC) of a single-crystal α-quartz grain slice. The matrix factorization in equation (29) has been used to reconstruct the strain field without the need to assemble the global system matrix. The top row ground-truth simulation input (GT) is to be compared with the middle row reconstructed strain field (REC). The bottom row shows the residual between reconstructed and true strain fields (RES).

**Figure 4:**
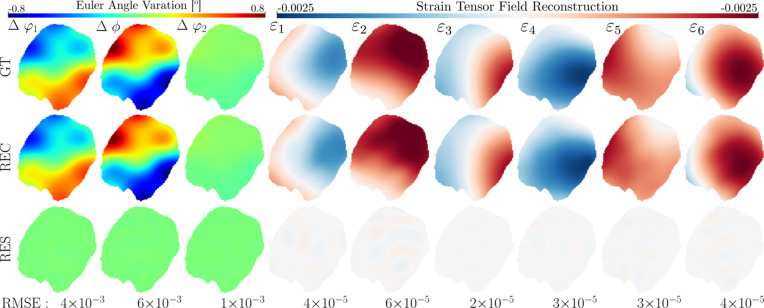
Coupled strain–orientation reconstruction (middle row) in a single slice of α-quartz. The simulated ground-truth (GT) field and corresponding data are described in Appendix *A* [App appa]. The residual field (RES) can be viewed in the bottom row. Note that the Bunge Euler angles (left) are displayed as a deviation from their respective mean values, allowing for a shared colorbar.

## Appendix A Demonstration example

To demonstrate the discussed matrix factorization in a practical application we have included a single-crystal X-ray diffraction simulation case study. Diffraction data were forward modelled from a 2D grain slice of α-quartz (SiO_2_) subject to a spatially varying strain tensor field,  , as well as a misorientation field,  , where  is the local crystal orientation matrix. The synthetic strain tensor field can be viewed in Fig. 2 ▸ together with the Bunge Euler angle variation, which was defined as

where  denotes volume average.

Using the space group of quartz (*P*3221) together with an X-ray wavelength of λ = 0.2846 Å, a total of 500 reflections were randomly (uniformly) selected in the Bragg angle interval θ = [4°, 13°] to be included in the simulation. Using the Laue equations,

where the matrix  maps from the integer reciprocal-space Miller indices,  , to crystal coordinates, diffraction vectors  were formed. The theoretical diffraction vectors were then corrupted with zero mean Gaussian noise as

where  for 90% of the measurements while  for the remaining 10%, emulating the presence of outliers. The 10% selected for outlier noising was selected randomly uniformly from the full diffraction data set.

Using the *ASTRA-toolbox* we have implemented the matrix factorization derived in this paper in operator format. This means that we never need to form the explicit sparse matrices involved in the forward model, but, instead, implement an on-the-fly operator that operates on an input vector to produce the system matrix vector dot product (and the corresponding adjoint product). This operator implementation is only possible thanks to the algebraic results that constitute the contribution of this paper. The code used to implement our matrix factorization (on an NVIDIA GPU architecture), produce the simulated data and reconstruct the strain (and later orientation) field is openly available as a demo Python library https://github.com/AxelHenningsson/flyxdm. In the same supplementary demo code, we provide detailed comments on the simulation and reconstruction setup and provide the code used to generate all figures found in these Appendices (including the generalizations to orientation fields made in Appendix *C* [App appc]).

## Appendix B Strain reconstruction

In this Appendix, we use the simulation data in conjunction with the Taylor expansion described by Henningsson & Hendriks (2021 ▸) and convert the diffraction vectors,  , into measurements of directional strain with the aim of reconstructing the intragranular strain field. In Appendix *C* [App appc] we describe how the diffraction vectors can be used without transform to reconstruct strain and intragranular orientation variations jointly, again exploiting our matrix factorization method. Note that while the aim in Appendix *B* [App appb] is to demonstrate our matrix factorization method for the case of strain reconstruction, the simulated data still originate from a grain that features both intragranular strain and orientation variation. As discussed elsewhere (Henningsson *et al.*, 2020 ▸; Henningsson & Hendriks, 2021 ▸), this is not a problem as long as the mosaicity of the grain is moderate.

In Fig. 3 ▸, the result of strain tensor reconstruction can be viewed. Here a radial basis expansion was used to construct  [in equation (29)]. The true noise covariance of the diffraction vectors was propagated through the Taylor expansion to construct the directional strain covariance yielding  . The small residuals and root-mean-squared errors (RMSEs) found in the bottom row of Fig. 3 ▸ are expected as a consequence of noise and model mismatch. For further details we refer the reader to the supplementary code.

## Appendix C Generalization to coupled orientation–strain models

We shall now consider generalizing our matrix factorization method to a diffraction model discussed by Henningsson *et al.* (2020 ▸, Section 6 equation 10-16). Considering the Laue equations (31) in integral form, we find

where  denotes volume average. The target intragranular field of deformation is now generalized to include the local rigid-body rotations as

In contrast to the small strain tensor,  ,  is not symmetric and the number of unknowns per point,  , in the grain is now increased to 9 (compared with six strain tensor components). To reach a similar factorization as that of (26), we start by introducing a flattened format of (33) as

where

and

and  is the  identity matrix. Let us now decompose the target field,  , on a finite basis as

where  are the scalar (pixel/voxel) basis functions and

Insertion of equation (38) into equation (35) yields after rearrangement that

where the linearity of the involved operators was used. We now note that equation (40) is a higher-dimensional copy of equation (10). Defining  according to equation (11), we find in analogy with equation (12) that

We now introduce a set of coefficient vectors,

and define a partitioned global coefficient vector as

The ray integral equation (40) can now be cast as

We now note that each row in equation (44) has an identical algebraic format compared with equation (15). Splitting the diffraction vector measurements into three separate vectors as

we are free to use the same arguments of row and column permutation as described in Section 3 to arrive at

where  now is a  block-diagonal projection matrix in direct analogy to equation (21) and the block-diagonal matrix  holds the Miller indices weighted by path length as

with

The global system of equations can now be written as

with

Alternatively, denoting the measurement vector as

and the Miller sampling matrix as

we arrive at our final factorized diffraction model,

The forward pass in equation (53) is defined by nine separate (scalar) projection operations followed by nine multiplications with the diagonal blocks (  ) of  . This factorization therefore admits the same computational benefits that are discussed for the decoupled strain model in the main paper. The discussion on generalizations held in Section 4, introducing a weight matrix  and a change of basis matrix  , is likewise applicable to equation (53). To verify that our derivations are correct we dedicate the following section to applying equation (53) to our demonstration example presented in Appendix *A* [App appa].

### c1. Application to demonstration example

For completeness we have implemented and solved equation (53) in our demo supplementary code for the same demonstration example that was considered in Appendices *A* [App appa] and *B* [App appb]. The same radial basis expansion as described in Appendix *B* [App appb] was used during regression. Likewise, the true noise covariance matrix was used to solve for the unknown radial basis coefficients in a weighted least-squares sense. The resulting maximum likelihood reconstruction (REC) can be viewed in the middle row of Fig. 4 ▸ together with the ground truth (GT) and residual (RES).
